# Two pediatric cases of alopecia universalis with concomitant atopic dermatitis successfully treated with baricitinib

**DOI:** 10.1016/j.jdcr.2025.05.039

**Published:** 2025-06-28

**Authors:** Shoichiro Muratani, Emi Sato, Shinichi Imafuku

**Affiliations:** Department of Dermatology, Fukuoka University Faculty of Medicine, Fukuoka, Japan

**Keywords:** alopecia areata, atopic dermatitis, baricitinib, pediatric dermatology

## Introduction

Alopecia areata (AA) is an autoimmune disorder characterized by nonscarring hair loss ranging from localized patches of hair loss to total scalp hair loss (alopecia totalis) or complete body hair loss (alopecia universalis).^1^ Although AA can affect individuals of all ages, pediatric cases create unique challenges because of the limited treatment options and potential psychological impact of hair loss during childhood.^2^^,^^3^ Pediatric AA, particularly the totalis and universalis subtypes, is often refractory to conventional treatments such as topical corticosteroids, excimer laser therapy, and contact sensitization therapy with squaric acid dibutylester.^1^^,^^4^, ^5^, ^6^

Baricitinib, a Janus kinase (JAK) inhibitor that targets JAK1 and JAK2, has been approved in the United States, Europe, and Japan for the treatment of severe AA in adults.^7^ Additionally, baricitinib has been approved for the treatment of moderate-to-severe atopic dermatitis in patients 2 years of age and older.^8^ However, baricitinib has not yet been approved for pediatric AA; therefore, its use for AA in the pediatric population remains off-label. We report 2 pediatric cases of alopecia universalis that were complicated by moderate atopic dermatitis and treated with baricitinib, which resulted in successful hair regrowth.

## Case report

### Case 1

A 9-year-old girl with a family history of atopic dermatitis (father) and allergic rhinitis (mother) presented with comorbid moderate atopic dermatitis, chronic urticaria, epilepsy, and trypanophobia. One month after contracting COVID-19 at age 7 years, she began experiencing hair loss. At 2 months after alopecia onset, excimer light therapy and topical corticosteroid treatment were initiated; however, disease progression continued. She had first presented to our hospital at age 8 years because nearly all of her scalp hair, eyebrows, and eyelashes were absent. Excimer laser therapy and topical corticosteroids were initiated; however, the condition remained resistant to treatment, and hair loss progressed to 100% within 6 months.

**Fig 1:**
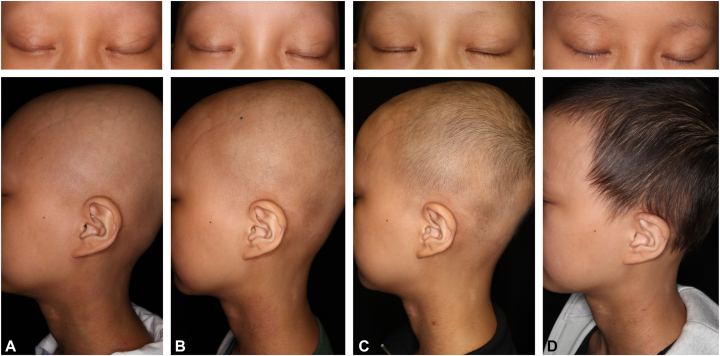
Alopecia universalis: clinical images of case 1. Clinical images obtained at **(A)** baricitinib treatment initiation and **(B)** 1 month, **(C)** 2 months, and **(D)** 4 months after baricitinib treatment initiation.

Short-term systemic corticosteroid therapy was considered, but the patient and her mother expressed significant concerns about its potential adverse effects on growth; therefore, it was not initiated. Because her atopic dermatitis was poorly controlled (Eczema Area and Severity Index [EASI] score, 16.0; Investigator Global Assessment [IGA] score, 3), dupilumab, which requires a high frequency of injections, was recommended. However, because of the patient’s trypanophobia, dupilumab was ultimately rejected. Therefore, oral baricitinib (4 mg), which has been approved for pediatric use, was initiated for our patient (body weight, 33.9 kg) (Fig 1, *A*). One month after baricitinib was initiated, atopic dermatitis improved (EASI score, 3.6; IGA score, 1), and hair regrowth occurred, thus reducing the hair loss area to 90% (Fig 1, *B*). At 2 months after baricitinib initiation, the hair loss area was further reduced to 40% (Fig 1, *C*);. At 4 months after baricitinib initiation, hair loss patches were no longer observed (Fig 1, *D*). At 6 months after baricitinib initiation, the patient’s condition was good, without any significant adverse events or AA recurrence, and an IGA score of 0 or 1 for atopic dermatitis was maintained.

### Case 2

**Fig 2:**
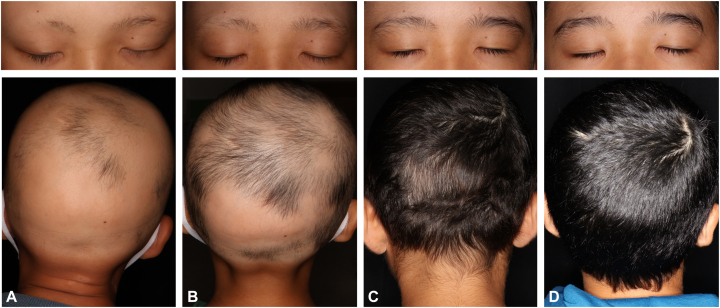
Alopecia universalis: clinical images of case 2. Clinical images obtained at **(A)** baricitinib treatment initiation and **(B)** 50 days, **(C)** 4 months, and **(D)** 7 months after baricitinib treatment initiation.

A 10-year-old boy with a family history of atopic dermatitis (younger brother) presented with comorbid allergic rhinitis and atopic dermatitis. Hair loss initially appeared on the frontal scalp 2 years before presentation and gradually progressed to the temporal and occipital regions, eventually resulting in total scalp hair loss and the loss of eyebrows and eyelashes. Excimer laser therapy was initiated, and complete hair regrowth was observed 5 months thereafter; therefore, treatment was discontinued. However, 1 month after discontinuation, rapid hair loss reoccurred and progressed. Because of the relapse severity, oral prednisolone 15 mg/day was administered and initially stabilized the disease. Nevertheless, after tapering and discontinuing prednisolone, the patient’s condition worsened again, resulting in total scalp hair loss. Therefore, excimer laser therapy was continued for 1 year; however, 80% to 90% hair loss continued. Therefore, contact sensitization therapy with squaric acid dibutyl ester and excimer laser therapy were initiated; however, this treatment strategy was discontinued after 2 months because of worsening atopic dermatitis (EASI score, 18.0; IGA score, 3). Therefore, the patient (body weight, 35 kg) was administered baricitinib 4 mg (Fig 2, *A*).

At 1 month after baricitinib initiation, atopic dermatitis improved (EASI score, 6.5; IGA score, 1). At 50 days after baricitinib initiation, hair loss improved to 50% (Fig 2, *B*). Complete regrowth of scalp hair was achieved after 4 months of treatment (Fig 2, *C*); therefore, baricitinib 4 mg was reduced to 2 mg. However, 3 months after the dose was reduced, the patient reported increased hair shedding. A direct examination indicated no apparent patches of hair loss, and the pull test yielded negative results (Fig 2, *D*). However, both the patient and his mother were concerned about potential relapse; therefore, the baricitinib dose was increased to 4 mg. At 10 months after dose escalation, hair loss and significant adverse events were not observed, and an IGA score of 0 or 1 for atopic dermatitis was maintained.

## Discussion

Both of our pediatric patients with alopecia universalis and concomitant moderate atopic dermatitis experienced significant hair regrowth within 4 months of initiating baricitinib treatment. Serious adverse events were not observed, and treatment was well-tolerated. Interestingly, the BRAVE-AA1 and BRAVE-AA2 trials, which involved adult patients with AA, reported that the Severity of Alopecia Tool (SALT) score at week 16 of baricitinib 4 mg treatment changed approximately 30% from baseline.^7^ Our pediatric patients achieved complete (100%) hair regrowth within 16 weeks of initiating baricitinib 4 mg. Rapid hair regrowth experienced by our pediatric patients highlights the importance of early intervention and suggests that baricitinib may be particularly effective for pediatric patients because of physiological differences in the hair cycle of children and adults and relatively higher drug exposure attributable to the body weight of our patients. The human hair cycle comprises the anagen (growth), catagen (transition), and telogen (rest) phases. As individuals age, the proportion of hair follicles in the anagen phase decreases, and more follicles enter the telogen phase.^9^ This shift is attributed to alterations in key signaling pathways, such as the Wnt/β-catenin, bone morphogenetic protein, and fibroblast growth factor pathways, which regulate the initiation and maintenance of the anagen phase.^9^ Children experience a higher proportion of remaining hair follicles in the anagen phase, potentially facilitating a faster response to JAK inhibition and more rapid hair regrowth when the inflammatory process is controlled. Additionally, pediatric patients have lower body weights than those of adult patients, which may lead to relatively higher systemic drug exposure per kilogram of body weight, thus enhancing the therapeutic effects of baricitinib and contributing to accelerated hair regrowth; moreover, a dose response has been observed during clinical trials of adult AA cases.^7^ However, a comparison of the efficacy of baricitinib for pediatric atopic dermatitis and that for adult atopic dermatitis revealed that the EASI 75 (the achievement of at least 75% improvement in the EASI score compared to that at baseline) rates at week 16 with concomitant steroid therapy for children and adults were comparable (4 mg: 47.7% vs 52.5%; 2 mg: 43.1% vs 40.0%). These findings suggest that the response of atopic dermatitis to baricitinib treatment does not significantly differ between adults and children. If baricitinib elicits a more rapid treatment response for pediatric AA than that for adult AA, then this difference is more likely attributable to disease-specific factors than to differences in drug exposure. Further phase III trials involving patients with AA are required to validate these observations.

Additionally, the treatment response of pediatric AA is heterogeneous and influenced by various factors. A meta-analysis of methotrexate treatment for AA indicated that adult patients have a higher complete response rate than pediatric patients (44.7% vs 11.6%), suggesting that the treatment responses of pediatric and adult populations may differ based on the therapeutic agent used.^10^

Early intervention for pediatric alopecia is important because hair regrowth can significantly enhance psychosocial well-being. Compared to adults, children are particularly vulnerable to the psychological impact of hair loss, which can lead to anxiety, depression, and a diminished quality of life. In school settings, children with noticeable hair loss may face social stigma, bullying, or exclusion, which can exacerbate emotional distress and hinder social development. Addressing alopecia promptly not only improves physical appearance but also fosters emotional resilience, thereby helping children navigate social challenges and maintain a positive identity.^2^

Although the usefulness of baricitinib for pediatric patients with AA was highlighted by these cases, it is not indicated for pediatric AA. Furthermore, the use of baricitinib is restricted to moderate or severe atopic dermatitis. Therefore, clinical trials that prove our observations and their generalization are necessary.

## Conflicts of interest

E.S. and S.I. received lecture fees from Eli Lilly Japan K.K. for topics related to atopic dermatitis and psoriasis.
